# A Ku-Band Miniaturized System-in-Package Using HTCC for Radar Transceiver Module Application

**DOI:** 10.3390/mi13111817

**Published:** 2022-10-24

**Authors:** Fan Yang, Bowen Zhang, Leijun Song

**Affiliations:** 1 Microwave Technology Research and Development Center, Beijing Institute of Radio Measurement, Beijing 100854, China; 2 School of Electronic Science and Engineering, University of Electronic Science and Technology of China, Chengdu 611731, China

**Keywords:** system in package (SIP), Ku-band, four channels, high temperature co-fired ceramics (HTCC)

## Abstract

This paper introduces a miniaturized system in package (SIP) for a Ku-band four-channel RF transceiver front-end. The SIP adopts the packaging scheme of an inner heat-dissipation gasket and multi-layer substrate in the high temperature co-fired ceramics (HTCC) shell with a metal heat sink at the bottom. The gasket effectively solves the heat-dissipation problem of high-power transceiver chips, and the multi-layer substrate achieves the interconnection between multiple chips. Within the limited size of 14.0 × 14.0 × 2.5 mm^3^, the SIP integrates five bidirectional amplifier chips, an amplitude-phase control multi-function chip, and two power modulation chips to realize the Ku-band four-channel RF transceiver front-end. Transmitting power over 0.5 W (27dBm) and receiving noise figure of 3.4 dB are achieved in the Ku-band. The efficient heat dissipation, high air tightness, and excellent integration are simultaneously realized in this SIP. The measurement results show that the performance is stable in the receiving and transmitting states, and the SIP based on HTCC technology has specific prospects for radar transceiver application.

## 1. Introduction

Ceramic material products have developed rapidly in recent years and have been widely used in many fields [1,2,3,4,5,6,7,8]. Ceramic package products have high packaging density, good electrical and thermal performance, and high reliability [9,10]. Compared with plastic and metal materials, they have obvious advantages in packaging, including good air tightness, high thermal conductivity, difficulty producing micro-cracks, and high-temperature tolerance. They also have broad application prospects in high-power micro-assembled circuits. High temperature co-fired ceramics (HTCC) [11,12,13,14,15,16] use high melting-point metals such as tungsten, molybdenum, manganese, etc. They are printed on aluminum oxide or an aluminum nitride ceramic green body according to circuit design requirements. About 6% of the sintering aid is then laminated in multiple layers and co-fired at a high temperature of 1600 °C. The typical processes include green tape casting, cutting, punching, hole-filling printing, lamination, firing, etc.

With the rapid development of integrated circuits, new materials, and packaging technology, active phased array radars are widely used in military and commercial applications. Transceiver modules are the key elements in many systems, and their size and weight are subject to severe constraints. This paper presents a Ku-band transceiver SIP module based on self-developed multi-function chips to meet these constraints. The module’s air tightness and heat dissipation are highly required in the radar transceiver system. Therefore, the SIP based on the ceramics package is appropriate for the radar application. Common types of ceramics packages are ceramic dual in-line package (CDIP), ceramic small outline package (CSOP), ceramic four-sided lead flat package (CQFP), ceramic pin grid matrix package (CPGA), ceramic ball grid matrix package (CBGA), etc. The ceramic package is widely used in numerous application fields, such as radio frequency filters (SAW, BAW), radio frequency integrated circuits (RFIC), optical communications, and sensors. That is based on its high dielectric properties, low loss characteristics, thermal expansion coefficient close to silicon wafers, and high structural strength.

The Quad Flat No-lead Package (QFN) is a widely used surface mount chip packaging technology with a small pad size and volume [17]. The QFN has excellent electrical and thermal performance because the large exposed pad in the bottom center is soldered to the carrier. The QFN is a leadless package, which is square or rectangular. There is a large-area exposed pad in the center of the package bottom, which affects heat conduction. There are also conductive pads for electrical connection on the periphery of the package at the bottom. The QFN package does not have gull-wing leads like the traditional small outline integrated circuit package (SOIC) or the thin small outline package (TSOP). Therefore, for the QFN, the conductive path between the internal pins and the bottom pads is short, and the self-inductance coefficient and the wiring resistance in the package are low. These characteristics give the QFN package outstanding electrical performance [18,19,20,21,22,23]. In addition, it also provides excellent thermal performance through exposed lead frame pads, which are direct thermal channels for dissipating heat within the package. Typically, thermal pads are soldered directly to the board, and thermal vias in the PCB help to dissipate excess power into the copper ground plane, which could absorb the excess heat. Due to its small size, light weight, and outstanding electrical and thermal properties, this package is ideal for applications in which size, weight, and performance are critical.

Although plastic packages can achieve lower costs, the products of the plastic package have significant defects in the reliability of packaging, internal thermal characteristics, storage, and application. Therefore, some devices need to be replaced by ceramic package products for several reasons: (a) package air tightness: plastic-packaged semiconductor devices are prone to inhalation of moisture; (b) limited internal thermal characteristics: different thermal expansion coefficients between plastic, frame, and chip; (c) limited application: plastic-packaged products are non-hermetic packages with poor thermal conductivity, which makes them unsuitable for high-power devices, and they are also greatly limited in aerospace and space applications. 

In recent years, many papers have reported high-performance Ku-band transceivers implemented by different integration methods. Some research results [24,25,26] used CMOS and SiGe semiconductor processes to achieve monolithic integration. Although the chip size was small, only a single-channel transceiver was realized, and the transmission power was less than 15 dBm. The authors of [27] achieved the Ku-band four-channel transceiver using the 180 nm CMOS semiconductor process. Every single channel transceiver with an on-chip antenna occupied 6.2 × 1.3 mm^2^ with a relatively high degree of integration, but the transmission power was only 14 dBm. The author of [28] adopted HTCC process technology to realize the broadband transceiver through SIP integration. The overall size of the SIP was 16 × 16 × 2.6 mm^3^. The bottom heat dissipation was carried out through an AlN ceramic substrate, but the transmission power and heat dissipation were not described in detail. The authors of [29] adopted LTCC process technology to realize single-channel SIP. The larger size of the SIP was 55 mm × 20 mm × 4.5 mm, which is not appropriate for the miniaturization integration of radar transceiver channels.

This paper uses HTCC package technology for high integration to realize a low-cost and miniaturized Ku-band multi-channel transceiver RF front-end with 0.5 W (27 dBm) transmitting power. Compared with all the above studies, this paper additionally integrates the pulse modulation function of radar transceiver switching in SIP. However, it is a crucial issue for miniaturized radar RF transceiver front-ends to achieve efficient heat dissipation, high air tightness, and excellent integration when packaged. This paper proposes a QFN package structure to reduce the package size effectively. By adopting a package solution in which a heat dissipation gasket and a multi-layer substrate are constructed in the HTCC shell with a metal heat sink at the bottom, efficient heat dissipation, high air tightness, and excellent integration are simultaneously realized. These are the main advantages of this SIP compared with other packages [30]. This SIP is suitable for implementing the radar transceiver system and engineering applications.

## 2. Materials and Methods

In the phased array design, the unit spacing of each transceiver module depends entirely on the operating frequency of the system. The designed working frequency of this module was 14.5–16.5 GHz in the Ku-band. At this frequency range, the spacing between the radiating units of each transceiver module was about 10 mm. Each unit sub-module had the same function and interface. Therefore, in the design, multiple channels are considered to be integrated with only one package, which could share the module’s power and control signals and improve the unit area’s use efficiency [31,32,33].

This paper provides a miniaturized system in package (SIP) for a Ku-band four-channel RF transceiver front-end for radar application [34,35]. The SIP integrates a variety of chips with different functions, including four bidirectional amplifier chips (BA1), one bidirectional amplifier chip (BA2), one amplitude and phase control multi-function chip (APCMF), one receiver power modulation chip (RPM), and one transmitter power modulation chip (TPM) [36]. These chips were integrated through the packaging solution of an inner heat dissipation gasket and multi-layer substrate in the HTCC shell with a metal heat sink at the bottom. The Ku-band four-channel RF transceiver front-end system was implemented within the limited size of 14.0 × 14.0 × 2.5 mm^3^.

**Figure 1:**
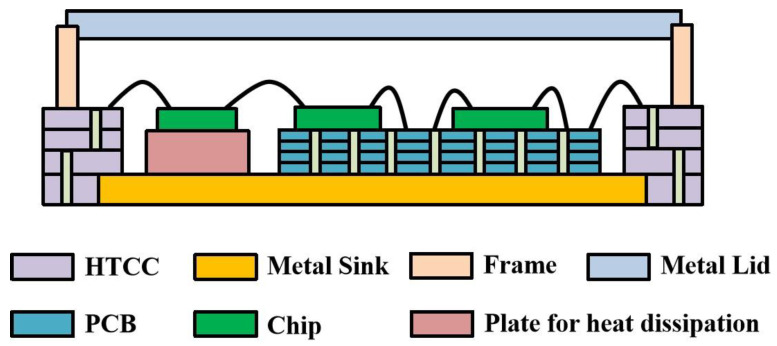
Diagram of the proposed SIP package structure.

The schematic diagram of the SIP structure is shown in Figure 1. The HTCC substrate with internal vias is at the edge of the QFN package, and the metal heat sink is at the bottom of the HTCC shell. Inside the package, the molybdenum copper plate was used below the power chip to conduct heat away, and the multi-layer printed circuit board (PCB) was used as the carrier of the power modulation chip, which is implemented by the CMOS process. The upper lid of the shell was made of iron–nickel alloy material with a thickness of 0.25 mm, except the edge thickness was 0.1 mm. The microwave monolithic integrated circuits in the SIP included five bidirectional amplifiers with a thickness of 0.1 mm, and an amplitude-phase multi-function chip with a thickness of 0.1 mm. The analog integrated circuits included two power modulation chips with a thickness of 0.3 mm. The heat dissipation plate and the multi-layer PCB were located on the metal heat sink of the package. Five bidirectional amplifier chips with heat dissipation requirements were mounted on the plate. Multi-function chips and two power modulation chips were mounted on the PCB. The chips and the shell were electrically connected through gold wires and the PCB.

The manufacturing process of the SIP is presented as follows: Firstly, the HTCC substrate was produced by green tape casting, cutting, punching, hole-filling printing, lamination, firing, etc. Secondly, the HTCC substrate, metal sink, and frame were welded together to form the HTCC shell. Thirdly, inside the shell, the plate for heat dissipation and the PCB were mounted on the metal sink by gold–tin welding. Fourthly, the power chips were mounted on the plate by eutectic soldering, and the other chips were pasted on the PCB by conducting resin. Fifthly, gold wires were bonded to connect the multiple chips and HTCC shell. Finally, the HTCC shell and metal lid were welded together to form a complete SIP.

**Figure 2:**
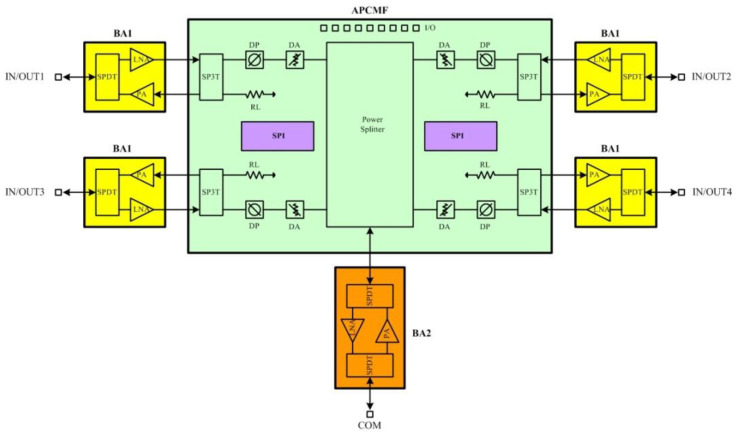
Schematic diagram of Ku-band four-channel transceiver SIP.

The system diagram of the SIP, which operates at 14.5–16.5 GHz, is shown in Figure 2. The Ku-band four-channel microwave chips were all implemented with a 0.25 um GaAs process, which has the advantages of lower noise figure and higher output power, including four BA1, one APCMF, and one BA2. The BA1 integrates a low-noise amplifier, a power amplifier, and a single-pole double-throw switch. The low-noise amplifier has a noise figure of 1.5 dB. The power amplifier has a saturation output power of 28 dBm and power-added efficiency of 38%. The single-pole double-throw switch has an insertion loss of 0.8 dB and high linearity characteristics, which has an output 0.1 dB compression point of 29.5 dBm. The BA2 integrates a low-noise amplifier (LNA), a power amplifier (PA), and two SPDT switches, which use the same PA, LNA switch as BA1. The APCMF integrates a serial-parallel conversion circuit (SPI), a four-way power divider, four 6-bit digital attenuators, four 6-bit digital phase shifters, and four single pole three throw switches. The SPI has the advantage of reducing the interface of control bits. The 6-bit digital attenuator has a 0.5 dB step size, 31.5 dB attenuation range, and attenuation accuracy RMS of 0.2 dB. The 6-bit digital phase shifter has a 5.625° step size and phase shift accuracy RMS of 3°. The single pole three throw switch includes three states of receive, transmit, and load. It is very convenient for single-channel measurement and performance evaluation. 

COM is the common port for transmitting and receiving, and IN/OUT is the transmitting and receiving port after the power is divided into four channels. The entire SIP works with a positive power supply of +5 V for BA1, BA2, TPM, RPM, and a negative power supply of −5 V for TPM and APCMF. The control logic of the GaAs switch is −5/0 V. The current consumption of +5 V power supply is 180 mA in the receiving state and 1.5 A in the transmitting state. The current consumption of −5 V power supply is 10 mA in the receiving state and transmitting state.

The four-channel RF transceiver front-end consists of multiple inner chips. Among them, the BA2 chip is at the common port, and the four BA1 chips are responsible for amplifying the receiving and transmitting signals of the four channels. The APCMF is responsible for the shift and attenuation of the radio frequency signal, transmission and reception of the control code, and the power combination or distribution of the four channels. The TPM realizes the gate and drain voltage modulation of power amplifiers in BA1 and BA2. The RPM realizes drain voltage modulation of low noise amplifiers in BA1 and BA2.

Transceiver SIP has two working modes: one is receiving mode, and the other is transmitting mode. When it works in the receiving mode, the microwave signal enters through the BA1, passes through the multi-functional chip for amplitude and phase control, and finally enters the BA2 for output. When it works in the transmitting mode, the microwave signal enters through the BA2 and passes through the APCMF. After the phase control of the APCMF, it finally enters the output of the BA1. 

## 3. Results and Discussion

### 3.1. SIP Design and Simulation

The ceramic material used in this SIP is aluminum oxide, which has a dielectric constant of 9.2 @16 GHz and a dielectric loss tangent of 1.5 × 10^−3^ @16 GHz. For this material, the breakdown field strength is more than 8000 V/m, and the insulation resistance exceeds 10^14^ Ω·cm. For other characteristics, the thermal conductivity of this material is 17 W/m·K, and the thermal expansion coefficient is 8 ppm/°C. Due to the low thermal conductivity, the heat dissipation was mainly through the metal sink at the bottom of the package.

**Figure 3:**
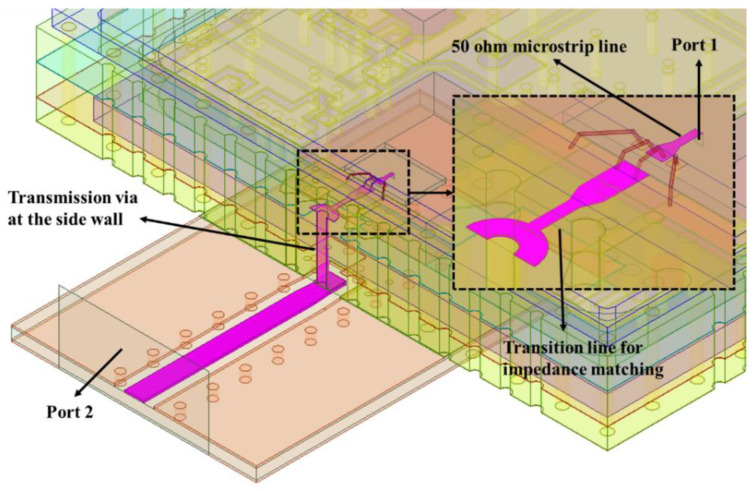
3D electromagnetic simulation of key microwave transmission structure of the SIP.

For electrical characteristics, to simulate the microwave performance of the SIP more accurately, first of all, it was modeled with high precision [37,38]. Based on the High Frequency Structure Simulator (HFSS) software for 3D electromagnetic field simulation, the model of the main microwave transmission path of the SIP was established, as shown in Figure 3. The transmission path was from the chip pad, bonding wire, HTCC shell (including the transmission via at the side wall) to the PCB board. The solution type in HFSS was set as driven modal. The port 1 for excitation was set as a lumped port, and the port 2 was set as a wave port. Between the port 1 and the signal pad of the chip, there was a microstrip line of 50 ohm and a tapered line. The transition line shown in Figure 3 was used for impedance matching. The diameter and spacing of the transmission vias at the side wall were also optimized to reduce impedance discontinuity. The size of the microwave signal pad was 150 × 100 µm^2^, and the bonding wire adopted two parallel wires with a length of 400 µm. The substrate of the microwave chip was set as gallium arsenide (GaAs) with a dielectric constant of 12.9.

**Figure 4:**
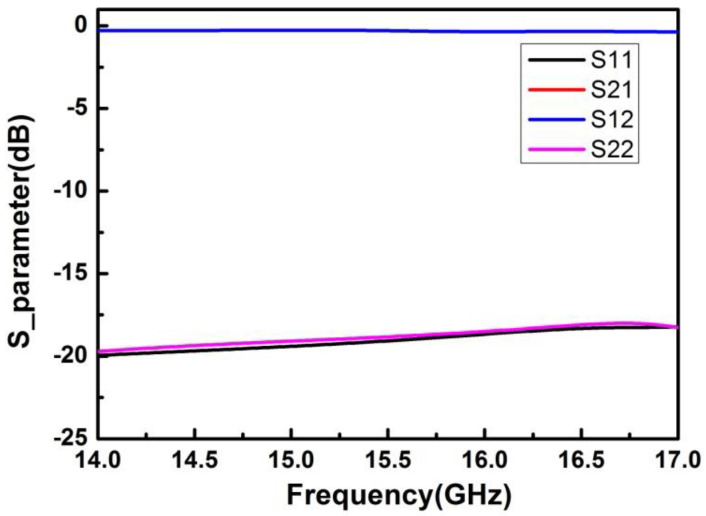
Simulation results of the S-parameters of the SIP.

The simulation results of S-parameters are shown in Figure 4. It can be seen from the results that the insertion loss (S21) of the microwave transmission path was about 0.3 dB, and the return loss (S11/S22) was less than −18 dB in the 14.5–6.5 GHz frequency range.

**Figure 5:**
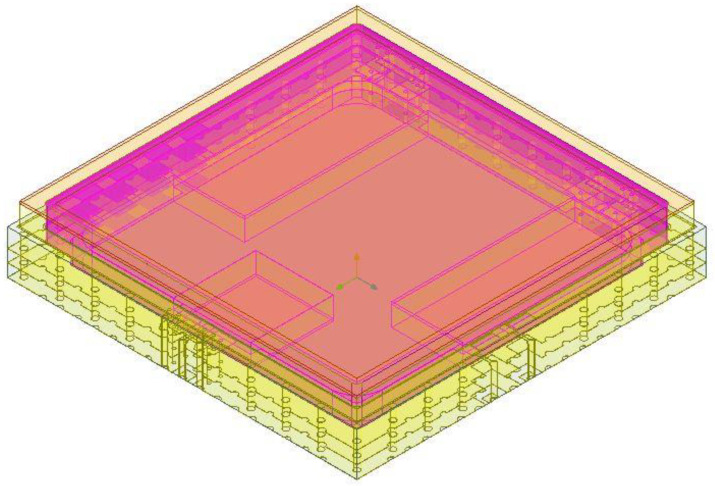
Cavity simulation model of the four-channel Ku-band transceiver SIP.

The HTCC shell and the metal lid need to be welded through parallel seam welding technology to guarantee the air tightness of the package. Consequently, there was a closed cavity inside the package where multiple chips were located. The substrate surface, interior, and through-hole pillars were all tungsten, and the surface was electroplated with nickel (Ni) and gold (Au) to meet assembly requirements such as soldering or wire bonding. The internal cavity of the transceiver SIP was simulated and analyzed to evaluate its influence. The simulation model is shown in Figure 5. Based on the High Frequency Structure Simulator (HFSS) software, the simulated frequencies and Q (quality factor) of the resonant modes inside the cavity are shown in Table 1. The Q is defined as [39]:

(1) $$Q=\omega \frac{\mathrm{average}\;\mathrm{energy}\;\mathrm{stored}}{\mathrm{energy}\;\mathrm{loss}/\sec\mathrm{ond}}=\omega \frac{\mathrm{Ws}}{\mathrm{Ploss}}$$

The ω is the angular frequency. The solution type in HFSS is set as eigenmode. We only selected the modes with resonant frequencies that are in the operating frequency range. From Table 1, we can see that the Q factors of these modes were at low levels, indicating that the modes will rapidly attenuate. Therefore, the influence of these modes can be ignored when analyzing the microwave performance of the SIP. 

**Figure 6:**
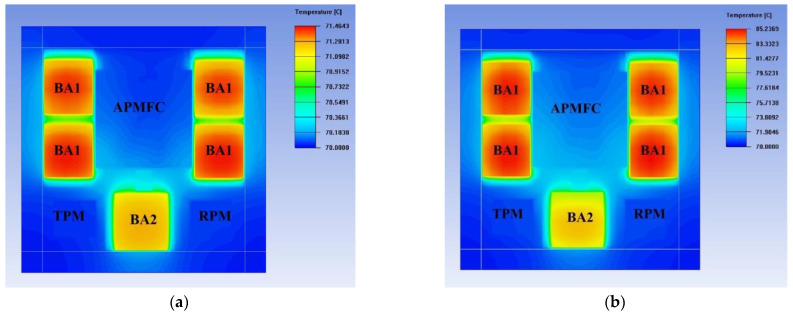
Simulation results of the thermal characteristic of the transceiver SIP. (**a**) The proposed SIP; (**b**) The SIP without a heat sink and gasket.

According to the demands of the working radar system, the maximum transmission pulse width was 3 ms, and the maximum duty cycle was 30%. The thermal characteristics of the SIP were simulated with the actual heat dissipation of the chips [40,41], as shown in Figure 6a. The simulation was based on the Ansys Icepak software. In the simulation, the package was on the plate with a fixed temperature of 70 °C, and the heat dissipation of the chips BA1 and BA2 was set as 1.7 W, respectively. For comparison, the result of another SIP without a heat sink and gasket is shown in Figure 6b, which means that all chips were mounted on the HTCC shell directly. The thermal resistances between the chips and the packages were computed to be 0.86 °C/W and 8.96 °C/W for Figure 6a,b. These results indicate that the SIP with a heat sink and gasket had a better ability for heat dissipation.

### 3.2. Measurement and Analysis

**Figure 7:**
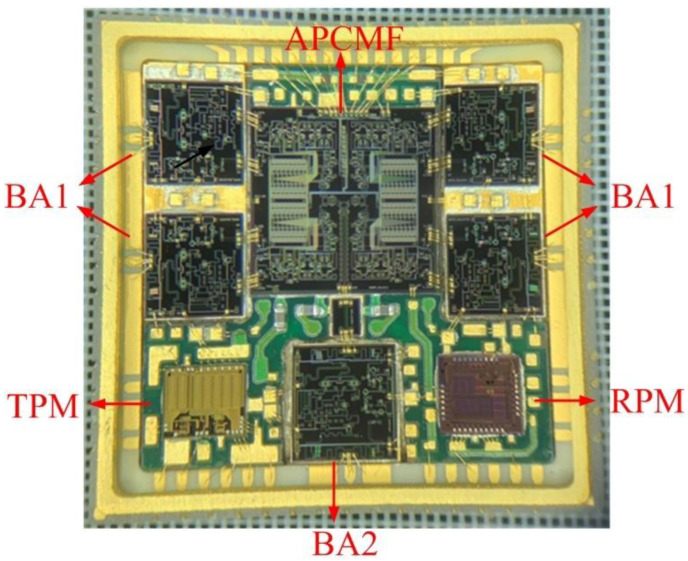
Internal photograph of the proposed transceiver SIP.

The photograph of the Ku-band four-channel transceiver SIP is shown in Figure 7. All microwave chips, power modulation chips, and several surface-mounted capacitors were integrated in the package. The outer dimension, including the frame and top metal lid, was 14.0 × 14.0 × 2.5 mm^3^. 

**Figure 8:**
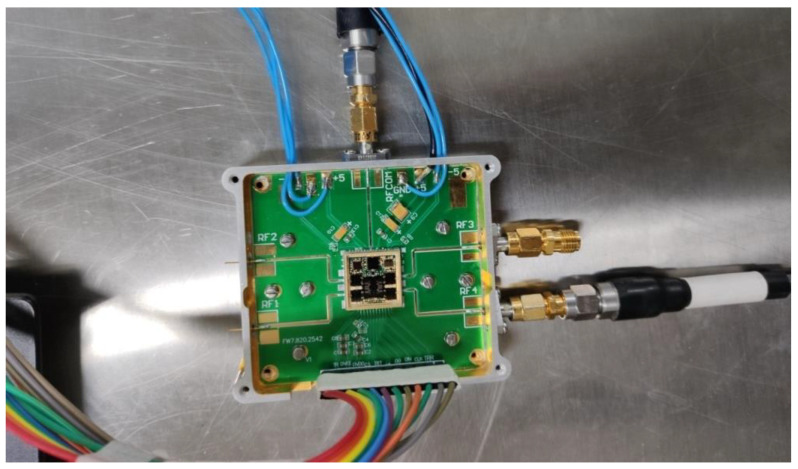
Test fixture of the proposed transceiver SIP.

The test fixture of the Ku-band four-channel transceiver SIP is shown in Figure 8. The SIP was mounted on the multi-layer microwave printed circuit board (PCB) by welding. External cables connected the power and control terminal to the test instrument. After the top metal lid was sealed by parallel sealing technology, the measurement shows that the air-tightness rate of this module was 9 × 10^−^^10^ Pa·m^3^/s.

**Figure 9:**
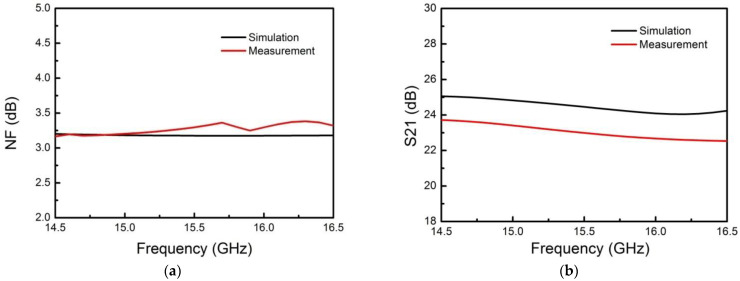
Comparison of simulation and measurement of the proposed Ku-band SIP in receiving state. (**a**) Simulated and measured noise figure curve; (**b**) Simulated and measured linear gain curve.

**Figure 10:**
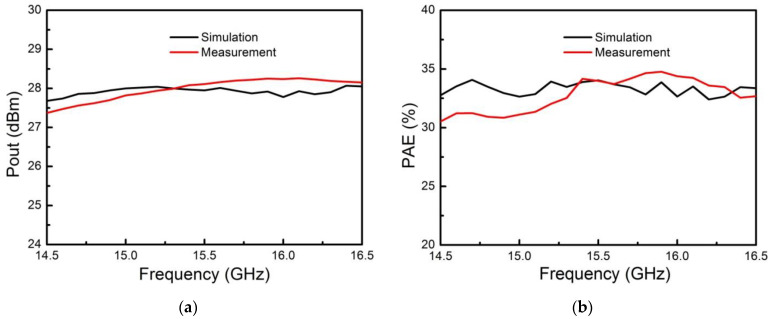
Comparison of simulation and measurement of the proposed Ku-band SIP in transmitting state. (**a**) Simulated and measured saturated output power curve; (**b**) Simulated and measured power added efficiency curve.

The vector network analyzer was used for testing to verify the actual performance of the proposed SIP. When testing in the receiving mode, the main test contents included receiving linear gain, noise figure (NF), output 1dB compression point, attenuation accuracy, phase shifting accuracy, and other indicators. When testing in the transmitting mode, the main test contents included saturated output power, power added efficiency (PAE), attenuation accuracy, phase shifting accuracy, and other indicators. The NF and linear gain curve in the receiving state are shown in Figure 9. The saturated output power and PAE curve under the transmitting state are shown in Figure 10.

It can be indicated from Table 2 that the SIP achieved the main functions of the Ku-band four-channel transceiver front-end. The attenuation and phase shift accuracy were very close between simulation and measurement because of the APCMF’s high-precision performance. However, there were still some differences between measurement and simulation results. In our opinion, the main factors resulting in the unsatisfactory measurement results were as follows: Firstly, the gold wires were difficult to bond at the periphery of the HTCC shell, which made it hard to control the shape and length of the bonding wires, thus introducing a specific insertion loss in the Ku-band. Secondly, for the transmission via hole at the side wall of the HTCC shell shown in Figure 3, there were differences between the actual dimension and the simulation model, and further optimization is needed. Thirdly, the crosstalk and interaction between multiple chips inside the package were not considered in detail in the simulation. When the SIP was working, the main crosstalk was between the multiple chips inside the package because the distance between the chips was so close, as shown in Figure 7. Filter capacitors were placed close to the power supply position of each microwave amplifier to reduce the signal crosstalk between the microwave chips. Moreover, the isolation of the power divider inside the four-channel APCMF was greater than 25 dB in the 14.5–16.5 GHz frequency range, which ensured that the amplitude and phase accuracy were less affected by adjacent channels. However, considering the intricate routes inside the PCB and other factors, the crosstalk of the SIP presented in the paper was more complicated and still needs further research.

## 4. Conclusions

A Ku-band four-channel transceiver SIP module based on HTCC technology was presented and verified. The efficient heat dissipation, high air tightness, and excellent integration were simultaneously realized in this SIP. The measured results prove that the SIP can work stably and satisfy the requirement of the system. This paper combined HTCC material, microwave monolithic integrated circuits, and miniaturized packaging technology to realize a Ku-band four-channel transceiver SIP for the miniaturization application of modern radar systems. Transmitting power over 0.5 W (27 dBm) and receiving noise figure of 3.4 dB were achieved in the Ku-band. Therefore, miniaturized SIP based on HTCC technology can be applied in large quantities in radar system transceiver components.

## Figures and Tables

**Table 1:** The simulated frequency and Q factors of the resonant modes inside the cavity.

Resonant Mode	1	2	3	4	5	6
**Frequency (GHz)**	14.66	15.18	15.37	15.66	16.22	16.55
**Q** **Factors**	186.28	189.43	393.15	187.15	192.16	191.76

**Table 2:** Measured results of the SIP’s key performance in receiver (RX) and transmitter (TX) mode.

Key Performance	Simulation Results	Measured Results	Unit
Operating frequency range	14.5–16.5	14.5–16.5	GHz
RX linear gain	24.0	22.5	dB
RX 1dB compression output power	13.2	12.7	dBm
RX noise figure	3.2	3.4	dB
TX linear gain	35.2	33.9	dB
TX saturated output power	27.6	27.3	dBm
TX power added efficiency	32.8	30.5	%
RX/TX attenuation accuracy	0.2/0.2	0.2/0.2	dB
RX/TX phase shift accuracy	2.0/2.1	2.3/2.5	°

## Data Availability

Not applicable.
